# Giant squamous cell carcinomas of the shoulder

**DOI:** 10.11604/pamj.2020.36.215.11644

**Published:** 2020-07-27

**Authors:** Mounir Yahyaoui, Najib Abbassi, Abdelhafid Derfoufi, Abdelkarim Daoudi, Omar Agoumi, Hicham Yacoubi, Abdeljaouad Najib, Badr Serji, Tijjani Elharroudi, Nabil Mouzouri, Siham Dikhaye, Nada Zizi, Hanane Hadjkacem

**Affiliations:** 1 Trauma-Orthopedics Service, University Hospital Mohammed VI, Oujda, Morocco,; 2 Oncology Surgery Service, University Hospital, Mohammed VI, Oujda, Morocco,; 3 Dermatology Service, University Hospital Mohammed VI, Oujda, Morocco; 4 Radiology Service, University Hospital Mohammed VI, Oujda, Morocco

**Keywords:** Squamous cell carcinomas, giant, shoulder, resection, reconstruction

## Abstract

Giant squamous cell carcinomas (SCC) larger than 5 cm in diameter are uncommon; there is no guideline on the size of an SCC that is considered giant. Treatment may be difficult with the need for large tissue resections and complex surgical reconstruction. We report a rare case of giant squamous cell carcinoma of the shoulder attached to deep anatomic planes. The entire mass was removed, resulting in a large defect that was repaired with myocutaneous flap rotation of the latissimus dorsi. Three courses of radiotherapy were performed after surgery. Fifteen months after the operation, the patient is well and working without any local recurrence and metastasis.

## Introduction

Squamous cell carcinoma (SCC)is the second most common skin malignancy [1]. It is prevalent in men and increases with age. The prevalence and incidence of SCC is increasing because of longevity and increased UV exposure associated with changes of lifestyle [2]. The larger and the deeper it grows, SCC is more likely to become metastatic [3]. Cutaneous SCC is an invasive and destructive tumor who is associated with a higher risk of disfigurement, local recurrence, and metastasis. We report the case of a patient with a giant SCC of the shoulder followed for 24 months post treatment without signs of local recurrence and/or metastasis.

## Patient and observation

**Figure 1:**
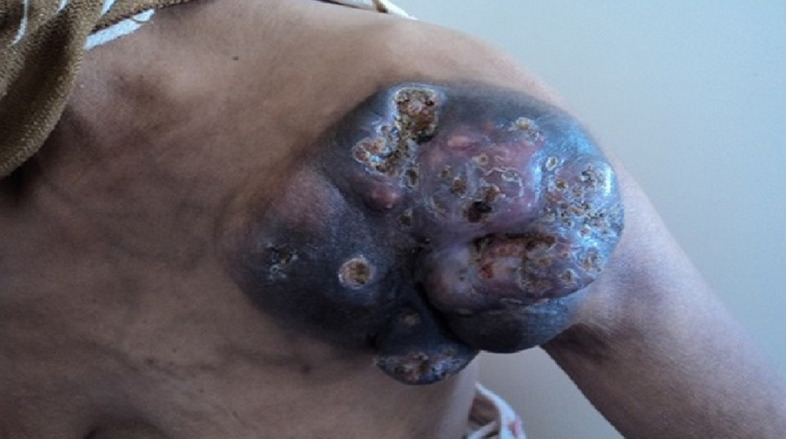
a 12x8-cm solid nodular mass on the left shoulder with infiltrated and exophytic appearance

**Figure 2:**
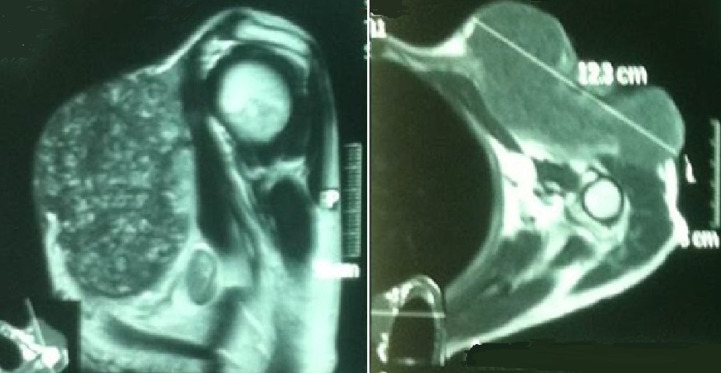
the mass invaded into deep anatomic planes

**Figure 3:**
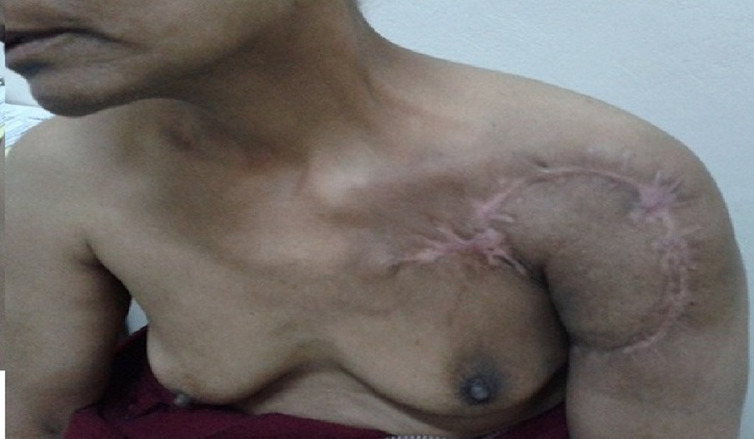
six months after excision of the tumoral lesion and coverage with myocutaneous flap rotation of the *latissimus dorsi*

A 50-year-old woman presented with a mass on her shoulder that had been increasing in size over the last 20 years. Physical examination revealed a 12x8cm extensive tumor of infiltrated and exophytic appearance (Figure 1), covered by necrotic material that was attached to deep anatomic planes (Figure 2). Staging showed no lymph node involvement or metastasis. The specimens revealed a diagnosis of well differentiated squamous cell carcinoma. The lesion was surgically removed with lateral margins of 2.5 cm. On excision, infiltration of the local muscles (pectoralis major, pectoralis minor and trapezius) was observed. All compromised structures were removed. The entire mass was removed, resulting in a large defect that was repaired with myocutaneous flap rotation of the latissimus dorsi (Figure 3). Histopathology showed findings that were consistent with well-differentiated SCC without perineural invasion and with clear surgical margins. Three courses of radiotherapy were performed after surgery. Fifteen months after the operation, the patient is well and working without any local recurrence and metastasis.

## Discussion

SCC accounts for approximately 20% of cutaneous malignancies [3] and occurs predominantly in elderly fair-skinned men. It has been shown to develop more frequently in chronically precursor lesions, including long-standing ulcers, sinus tracts, burns or osteomyelitis [4]. The incidence of SCC has increased considerably over the past 20 years, because of the growing life expectancy, and increased sun exposure [5]. A history of exposure to sunlight during childhood may be the greater risk factor because of the harmful effects of ultraviolet exposure [6]. SCC can show de novo onset or more often mark the progression of several precursor lesions: actinic keratosis and Bowen disease [5]. Among patients with invasive SCC, multivariable analysis showed a significantly higher likelihood of large size for SCC arising on chronic lesions and treatment delay longer than 1 year, and for SCC on not easily visible sites, it has reported a lower likelihood of significant association between larger lesions and anatomic site [7]. Giant SCC larger than 5 cm in diameter are uncommon; there is no guideline on the size of an SCC that is considered giant [8]. To our knowledge, there was no report of SCC case in this location treated by such a very extensive resection. Unexpectedly, no metastasis and local recurrence have been observed for long. It may suggest that radiation therapy and extensive operation we performed was effective treatment for such a huge SCC. In the majority of cases, large SCC is associated with a higher risk of disfigurement significant tissue destruction, requiring major plastic surgery with a potentially positive impact on morbidity, mortality, and costs.

Tumor diameter is a major prognosis criterion. Lesion size ≥ 4 cm and histologic evidence of perineural and deep invasion into the deeper tissues were the clinical-pathologic factors associated with higher rates of local recurrence and regional metastasis and lower rates of survival [9], they carry twice the risk for recurrence and 3 times the risk for metastasis [3]. Early studies have shown that whilst both tumour diameter and tumour thickness are independent risk factors for metastatic only the latter is an independent risk factor for local tumour recurrence [10], actually risk factors associated with metastasis include also histologic grade, location, recurrence, and immunosuppressed state [3]. Histological-poor prognostic signs include a desmoplastic growth type, tumor thickness of 3.5 mm, only with invasion depths of Clark level IV or more did recurrence take place and metastatic spreading only occurred from Clark level V [11], the other factors were ulceration, poor differentiation, and over ten mitoses/3 HPF 400 on the tumor growth front [12]. Biopsy is done to confirm preoperative diagnosis before extensive surgery and if the clinical diagnosis is uncertain [5]; surgical excision remains the gold standard with oncological complete excision and histologically tumor free margins [8]. SCC should be appropriately excised with surgical margins of 4 to 10 mm, depending on the size of the tumor [10]. Surgical margins of at least 6 mm are needed to achieve healthy margins in tumors with a diameter greaterthan 2 cm. The judgement of where a tumor ends and when a deep plane is clean can be complicated by local anatomy, however Tumor thickness and depth of invasion are important prognostic factors in SCC [11], a better deep clearance may be achieved by excising an extra deep fascial plane respecting fascia, periosteum, and perichondrium because these structures are not in direct contact with or invaded by the tumor [13].

Nevertheless, Mohs micrographic surgery remains an interesting treatment for these larger tumors and high-risk SCC [11]. Conventional surgery has a higher cure rate than Mohs surgery overall, but it is less successful with tumors that poorly differentiated, or recurrent [11]. Appropriate reconstruction methods should be based on the location and size of the defect created after tumor excision, in our patient flap rotation of the latissimus dorsi were satisfactory options for reconstruction. Radiotherapy may be considered for cases in which tumor cells are close to surgical margins or in which negative margin control is not possible [1]. One study of 167 patients with SCC and perineural invasion revealed a local recurrence rate of 43% with surgical excision alone compared with 20% with excision and adjunctive radiation. Likewise, disease-free survival increased from 53% to 73% in the 2 groups, respectively [14], they conclude that a more conservative surgical approach followed by adjuvant radiotherapy may be a reasonable way.

## Conclusion

Squamous cell carcinomas that reach large dimensions are accompanied by the risk of disfigurement, local recurrence, and metastasis. Aggressive treatment is the best way to reduce the morbidity and mortality associated with this disease.
